# Preparation of IMGs for Residency Training in Canada

**Published:** 2012-03-31

**Authors:** Andrew Duncan, Megha Poddar

**Affiliations:** McMaster University, Hamilton, Ontario, Canada

International medical graduates (IMGs) are becoming an increasing proportion of the medical workforce in Canada; the number of IMG-funded postgraduate training positions has more than tripled since 1999 (from 291 to 1065).^1^ IMGs seeking Canadian residency positions have diverse nationalities and provide a potential wealth of knowledge and experience to the Canadian medical system.

Much has been said regarding the difficulties securing a Canadian residency position for IMGs, but there has been little expressed regarding assimilation in to the Canadian medical system for successful applicants. Because foreign medical schools are intrinsically different, the initial integration into the Canadian system for incoming IMG residents can be extremely challenging. For many foreign medical programs, the equivalent of the Canadian clerkship period takes place in the intern year, after they have qualified as physicians. As such, IMG physicians who are accepted to Canadian residency programs prior to completion of their intern year often have had little exposure to clinical responsibilities (i.e. dictations, progress notes, discharge summaries, etc.). Given this, incoming residents who come from not only a different medical program, but also a different medical system, may be at a significant disadvantage compared to their Canadian-trained colleagues.

In Ontario, the assimilation of IMGs into the workforce is achieved through a three-week orientation program offered by the Centre for the Evaluation of Health Professionals Educated Abroad (CEHPEA) entitled The Orientation to Training and Practice in Canada Program (OTPC). This program focuses largely on appropriate and effective delivery of communication skills and provides an introduction to the framework of the Canadian medical system and culture.^2^

A recent survey, distributed to 40 first and second-year IMG residents in primary specialty programs at McMaster University, highlighted that only 17.9% of respondents felt that the CEHPEA program adequately prepared them for residency in a Canadian medical system. Furthermore, the survey revealed that over 90% of respondents felt that practical training with regard to resident duties would be extremely beneficial prior to starting their residency. Finally, when asked to rank the most challenging obstacle to starting a residency in Canada, the overwhelming response was “the carrying out the day-to-day responsibilities of a resident.” Sub-group analysis showed that the answers were similar for both Canadians who Studied Abroad (CSAs) and non-Canadian IMGs.

As a comparison, Canadian Medical Graduates (CMGs) encompassing various specialties were also provided with the survey (n = 30). Compared to their IMG colleagues, 93% of CMGs felt well to extremely well prepared prior to commencement of their residency. Additionally, over 90% of respondents felt “comfortable” to “very comfortable” with the day-to-day responsibilities of Canadian residents (admission notes, dictations, etc.) Furthermore, only a third of CMG respondents felt that additional training in core resident duties would be beneficial, compared to over 90% of IMGs. Taken together, it is clear that CMGs entering their residency are much better prepared for the rigors of life as a resident in a Canadian medical system compared to their IMG counterparts.

At McMaster University, we have identified a crucial gap between what is expected of residents and the comfort level of incoming IMG residents. Our research shows that IMG residents did not feel adequately prepared for life as a Canadian resident compared to their Canadian-trained colleagues. In Ontario, it would be prudent to re-examine the way IMGs are assimilated into the Canadian medical system, with a focus on bridging this gap.
